# Improving health and sustainability: Patterns of red and processed meat consumption across generations

**DOI:** 10.1016/j.healthpol.2022.10.006

**Published:** 2022-12

**Authors:** Cinzia Di Novi, Anna Marenzi

**Affiliations:** a European Commission, Joint Research Centre (JRC), Via Enrico Fermi 2749, TP 267, Ispra, VA 21027, Italy; b Department of Economics, Ca’ Foscari University of Venice, San Giobbe, Cannaregio 873, Venice 30121, Italy

**Keywords:** Red meat, Processed meat, Health, Sustainability, Environmental impact, Generations

## Abstract

This study explores whether the widespread dissemination of Western-type culture and the globalization of food production and consumption that have characterized Italy for decades may have influenced red and processed meat consumption across generations. For the purpose of our study, we constructed a pseudo-panel derived from repeated cross-sections of the annual household survey, “Aspects of Daily Life,” that was part of the Multipurpose Survey carried out by the Italian National Statistical Office (ISTAT) from 1997 to 2012. We adopted an APC (Age, Period, Cohort) approach that involves age, period, and cohort effects. We followed the experiences of four cohorts: the Silent Generation (born 1926–1945), the Baby Boomer 1 Generation (1946–1955), the Baby Boomer 2 Generation (1956–1965), and Generation X (1966–1980). Our results revealed that increases in disposable income, changes in women's role in society, and urbanization and globalization have had significant effects on consumption patterns. The analysis shows that the older generations have changed their diets more in favor of meat consumption than later generations, with more change in the relatively affluent north of the country compared with the south, while the youngest generations are more likely to adopt more healthful and environmentally sustainable eating patterns.

## Highlights

• This study investigates red and processed meat consumption across generations in Italy.
• The widespread dissemination of Western-type culture have reduced adherence to the mediterranean Diet.
• The older generations have changed their diets more in favor of meat consumption.
• The youngest generations adopt more healthful and environmentally sustainable eating patterns.

## 1 Introduction

Food-consumption habits are undergoing profound changes. The last century has shown a conspicuous increase in meat consumption, thanks also to economic growth, developments in meat production technology and intensified urbanization. However, meat production and consumption are also subject to criticism for environmental and health-related reasons. The production of meat has become a major concern because of the impact of livestock on global warming and environmental degradation [1], [2], [3], as according to the Food and Agriculture Organization (FAO) [4], in 2013 livestock farming accounts for a significant proportion (14.5% in 2013) of anthropogenic greenhouse gas emissions. High consumption of meat, especially red met, has also been associated with a risk of diet-related non-communicable diseases (“chronic disease” hereafter). Meat is a good source of energy and some essential nutrients, including protein and micronutrients like iron, zinc and vitamin B12, but its consumption is a risk factor for cardiovascular disease because of its saturated fat and cholesterol content. In the 2007, the World Cancer Research Fund and the American Institute of Cancer Research (WCRF/AICR) published a report based on a systematic review of previous research and concluded that high intake of red and processed meat (i.e. meat that has been transformed through salting, curing, fermentation, smoking, or other processes to enhance flavor or improve preservation) convincingly increases the risk of colorectal cancer [5]. In October 2015, the World Health Organization International Agency for Research on Cancer (IARC) announced that consumption of processed meat is “carcinogenic to humans” (Group I) and that consumption of red meat is “probably carcinogenic to humans” (Group 2A).

In the food context, reducing the environmental impacts of food consumption requires a shift to the “plant-based” diets that require less land, energy, and other resources than meat-based diets do [6]. One of the best-studied examples of a diet that emphasizes plants and is consistent with environmental sustainability is the Mediterranean diet (MD) (i.e., the traditional dietary pattern of people living in the Mediterranean basin). Adherence to the MD has been associated historically with longevity, lower prevalence of chronic diseases, and a significant reduction in mortality from cardiovascular disease and cancer ([[7], [8], [9]]; Sofi et al., 2018).

However, the widespread dissemination of Western-type culture and the globalization of food production and consumption have reduced adherence to the MD in the Southern European countries where it originated [10]. This decline has two main aspects: increased consumption of refined grains, saturated fats, sugars, and red and processed meat and decreased consumption of complex carbohydrates like cereals and legumes [11]. Social changes, too, appear to have contributed to the shift in dietary habits in Southern European societies: Changing household structures (with more single households), women's participation in the labor force, longer working hours, and consumer prosperity may also have opened the door to such ready-to-eat and convenience food as processed meat [[12], [13], [14]]. Thus, the influence of Western-type culture and social changes may have seriously threatened the transmission of the MD heritage to the youngest generations, despite their increasing sensitivity to environmental and health-related issues.

This study explores whether the changes in the technology, culture and social welfare that have characterized Italy for decades may have influenced red and processed meat consumption across generations. We followed the experiences of four cohorts: the Silent Generation (born 1926–1945), the Baby Boomer 1 Generation (1946–1955), the Baby Boomer 2 Generation (1956–1965), and Generation X (1966–1980).

The Italian context is similar in many ways to those of other countries in Southern Europe, but Italy underwent a dramatic change in the second half of the last century, passing from an agro-familial society to an industrialized society. This change produced a significant shift in food habits, increasing animal product consumption, particularly meat [15]. Among Southern European countries, Italy ranks medium-high in terms of red meat and processed meat consumption [16]. Differences in the dietary transition in Italy are also related to geography in terms of areas that have relied on agricultural production but also on differences in the degree to which Western-type culture has penetrated [17]. These peculiarities make Italy a particularly interesting case study.

The results revealed that the Silent and Baby Boomer 1 generations shifted from the traditional MD in terms of red meat consumption and that the Baby Boomer 2 generation shifted in terms of processed meat consumption. Our findings also confirm that the youngest generations are the most adherent to the MD, thanks to their higher sensitivity to the environmental and health-related impacts of meat production but also to their concern about the .animal rights and the rearing conditions of the farm animals. Indeed, according to the latest data from the EURISPES [18] report, in Italy the proportion of people who are adopting a vegetarian diet is constantly increasing especially among those who are younger. About 7% of the Italian population aged 35–44 declared to be vegetarian in 2019, against about 5.8% of the population aged 45–64 and 4.7% of the over 65 .

The empirical results lead to the need for reflection concerning the oldest generations. Older age is typically associated with worse health, more use of the healthcare system and increased healthcare costs, so the older generations’ high consumption of red and processed meat during early adulthood may present a significant and increasing challenge for today's National Health Service as these generations develop chronic health conditions, including cardiovascular disease and cancer.

The remainder of the paper is organized as follows. Section 2 describes the generational changes in food consumption across generations, the data and the empirical strategy, while the results are presented and discussed in Section 3. Concluding remarks are made in Section 4. The appendix contains additional material.

## 2 Material and methods

### 2.1 Context: generational changes in food consumption

An individual's eating regime encompasses more than just food; it is a social marker that, through its symbolic value, constructs social identities [19]. Food choice is a complex system influenced by multiple factors related to consumer preferences and to the consumption context: culture, economic conditions, and environmental and health concerns ([20,21]; Stasi et al., 2018). Among the motivations that influence food choices, the consumption context plays a major role and may explain the differences in food preferences across generations [[22], [23], [24], [25]].

Here, we review the diffusion of red and processed meat in Italy following the experiences of four cohorts: the Silent Generation (born 1926–1945), the Baby Boomer 1 Generation (1946–1955), the Baby Boomer 2 Generation (1956–1965), and Generation X (1966–1980).

We contextualized the consumption of red and processed meat according to the economic conditions and the cultural factors that characterized Italy in the decades since 1950. We identified the median age at which women of each birth cohort left their parental homes in the transition into early adulthood. (See Table 1A in the Appendix.) We focused on women because of their traditional role in preparing meals and food management [26]. We considered leaving one's parents’ home as a central event in the transition into adulthood [27], as it can disrupt parents’ diet and dietary behaviors and give the younger generation more autonomy in preparing meals and decision-making related to food [28]. We concentrate on early adulthood since behaviors established during this period, such as dietary intake and eating behaviors, tend to persist into later adulthood and to influence the risk of chronic disease in later life [29].

The Silent Generation entered early adulthood between the end of 1940s and the end of 1960s, a period during which Italy enjoyed intense economic growth. All of the macroeconomic variables -national GDP, GDP per capita, exports/imports, investments, and so on - grew to unprecedented levels and, in parallel, household spending leapt forward in terms of both the quantity and quality of food consumption. The increase in food consumption was significant (albeit much lower than that of other European countries), a remarkable transformation from the past, as a richer and more varied diet became accessible to classes that had not previously had access to it [30].

The conspicuous decline in the 1950s in the consumption of maize, rye and barley, lard and suet, legumes, and dried fruit, together with the conspicuous increase in the availability of fresh vegetables, fresh fruit, milk, and beef transformed Italy into a “sated” country that began to suffer from the negative health consequences of such a rich and abundant diet, including cardiovascular, endocrine and metabolic diseases [31].

Between 1950 and 1970, food consumption per household more than doubled as the richness of people's diets increased: the consumption of grains flattened out as meat consumption (especially beef) increased (see Fig. 1A in the Appendix).The result was a European diet that combined a high level of animal proteins with a MD of pasta, fruit and vegetables, of which Italy was among the main European producers, a combination that was, in many respects, unique in Europe. Calorie intake increased from an average of around 2350 calories per person in the early 1950s to 3000 calories in the late 1960s and 3200 in the following decade [32]. Italy ranked medium-high in terms of beef consumption among the countries of Europe (see Fig. 2A in the Appendix).

In the 1970s, Italy experienced a fundamental change in the daily consumption of the animal proteins that had been scarce in the past. With approximately 61 kg of meat consumption per capita per year at the end of the 1970s, Italy jumped to thirteenth place in consumer countries’ rankings, a significant leap forward compared to twenty years earlier, when it was at the bottom of the scale. This leap is even more significant when we look only at the ranking for beef consumption, as Italy rose to fifth in the world [32]. It was in this period that the Baby Boomer 1 generation began to enter early adulthood.

In the 1980s, the picture changed again. Much of the Baby Boomer 2 generation reached young adulthood in this period, which was characterized by improved economic wellbeing and faster technological innovation compared to the past. This was also the period of a progressive reduction in the consumption of wheat, wine, and sugar, stabilization in the consumption of red meat, and a further increase in that of fruits and vegetables. The consumption of red meat, especially beef, after a phase of strong expansion in the first twenty years after World War II, slowed down at the end of the 1970s, and lost additional ground in the following decade and later. However, increasing prosperity, technological changes, participation of women in the labor force (especially in the North of Italy) contributed to an increase in consumption of convenience foods like processed meat as time-saving meals.

In the new millennium, when much of Generation X entered adulthood, the consumption of poultry and pork increased significantly and overtook that of beef. The spread of the Bovine spongiform encephalopathy (BSE) epidemic certainly affected the consumption of beef. BSE, nicknamed “mad cow disease,” frightened consumers and redirecting them to alternative, cheaper meat while drastically cutting down beef consumption. Between the old and the new millennium, beef lost its appeal as a symbol of nutritional well-being and family wealth and became synonymous with an unbalanced, unhealthful and environmentally unsustainable diet, especially for the youngest generation. In the same period, the consumption of processed meat also decreased with a reassessment of dietary models linked to the MD.

### 2.2 Data and estimation strategy

We adopt an APC approach, which involves age, period, and cohort effects [33], for our analyses using a pseudo-panel derived from repeated cross-sections of the annual household survey, “Aspects of Daily Life,” which is part of the Multipurpose Survey system carried out by the ISTAT. The analysis uses fifteen years of repeated cross-sections from 1997 to 2012 (excluding 2004, when the survey was not fielded). In constructing the pseudo-panel, we divided each year's observations into cohorts (individuals who share some common characteristics) and used these cohorts to estimate a fixed-effects model from repeated cross-sections [34,35]. The main assumption behind the construction of a cohort is that it consists of respondents who share a set of characteristics that do not change (e.g., birth year, gender) or that remain broadly constant over time (e.g., region of residence), have similar food consumption habits, and can be treated as a single observation [36].

In choosing the width of the cohort, we aggregated birth cohorts based on the ISTAT definition of generations (Section 2) to assess the differences in red and processed meat consumption (“generation effects”). We followed the experiences of four birth cohorts: those born 1926–45 (the Silent Generation), 1946–55 (the Baby Boomer 1 Generation), 1956–65 (the Baby Boomer 2 Generation), and 1966–80 (Generation X).

Then we considered the trade-off between the need to have as much informative data as possible and the need to have a sufficiently large number of observations per cell to reduce the potential for error in estimating the cohorts’ means. A large number of observations in each cell helps to ensure that the necessary asymptotic theory is applicable to the pseudo-panel approach. The problem of the number of individuals in a cell can be ignored and cohort data can be treated as genuine panel data if the number of individuals in each cell is above 100 [36].

Our choice of cohorts included a pseudo-panel that was constructed on two genders, four birth cohorts, and two educational levels (those who left school at the compulsory age and those who undertook additional voluntary schooling (cf. [37])) in nineteen regions over fifteen years. The cut-off we used for the respondents’ educational level may seem crude and to involve significant loss of information, but this construction allows us to assume that education is a time-invariant characteristic since all of the participants included in the sample were old enough to have been able to complete at least the compulsory education. We did not consider tertiary education as a cut-off in splitting the sample since the percentage of respondents with tertiary education in the oldest generations, particularly the Silent Generation, was too low, especially among women (3.2%). Even though younger generations have seen an increase in their level of education, Italy's tertiary education attainment rate remains one of the lowest in the EU: according to the European Commission Education and Training Monitor [38], the average rate of tertiary education attainment in Italy is 28.9%  against 40.5% of the EU.

The literature has often interpreted food choices in relation to consumers’ socioeconomic characteristics: Higher education levels in particular have been associated with lower consumption saturated fats, sugars, and red and processed meat, while lower education levels have been associated with high-fat food and energy intake. It is likely that a high level of education stimulates information-acquisition behaviors, involvement with healthful foods, and a preference for natural and light foods, improving health and increasing health-oriented behaviors [39].

Finally, we averaged all of the variables over the year among individuals of the same gender and within birth cohorts, educational levels and regions to find a time series for each cohort [34]. The resulting average number of individuals per cell is 128 for the entire sample (Italy), 143 for the Northern regions, and 121 for the Southern regions (See Table 2A in the Appendix.)

### 2.3 APC models and “the identification problem”

From a methodological point of view, age–period–cohort models suffer from an identifiability problem because of the relationship between the variables: Year of birth plus age equal calendar year (cohort + age = period). Hence, unrestricted age, cohort, and period effects cannot all be separately identified [40]. The literature makes several attempts to find a solution to this problem, one of the most common of which is to constrain certain parameters in a model so they are equal. (See Bell and Jones [41] for details.) Each age and birth cohort group is included in a regression model as a dummy variable, but two age groups and cohort groups are combined into a single group (e.g., [42]). This approach solves the problem of perfect collinearity, but as Glenn (2005, p. 12) pointed out, “When this is done, the linear dependence is broken in the statistical model only and not in the real world [so] the obtained estimates of effects are not meaningful.”

We addressed the identification problem by employing Deaton's [34] solution, which decomposes temporal change into birth-cohort dummies and a continuous age profile, while period effects are regarded as exogenous shocks that sum to zero in the long run. This is the standard decomposition Deaton [34,35] proposed. Of course, other variables might influence red and processed meat consumption. For instance, a recent study by Sares-Jäske et al. [43], which considers the role of household composition, has shown that women living in household with children consume more red and processed meat than other women. However, the “women living in household with children” dummy variable at the individual level becomes “the proportion of women who live in household with children in the cohort c on the date t” in the pseudo-panel data. Deaton [35] did not recommend the introduction of these types of variables in the analysis.

In keeping with on Bell and Jones [41] and Veday [44], we assumed that changes in red and processed meat consumption over time is the result of birth cohorts’ differing attitudes toward red and processed meat, rather than period effects, and allocated temporal trends to variations in age or cohort. (See also Veday [44]). We estimated a separate regression equation for consumption of red meat and processed meat.

Concerning the dependent variables (red meat consumption adherence to MD and processed meat consumption adherence to MD), we followed Benedetti et al. [45] in constructing an index (a composite score) based on the Mediterranean pyramid recommendations to evaluate the Italians’ adherence to the MD with reference to red meat and processed meat [[46], [47], [48]]. The Multiscopo Survey includes a section that is devoted to the exploration of individuals’ food-consumption habits, where the respondents report the frequency of their intake of red and processed meat in terms of times per day and week. We assigned a score ranging from 0 to 4 to each respondent's frequency of consumption based on the degree of adherence to the MD with the score ranging between 0 (lowest adherence) and 4 (highest adherence) (Table 1).

**Table 1:** Scores for meat group.

Meat Group	More than once per day	Once a day	A few times per week	Less than once a week	Never
Red Meat	0	0	2	4	3
Processed meat	0	0	1	4	3

Then, using cohort data, we transformed the dependent variable into the mean value of the score in the birth cohort, for each gender, with two level of education, in each region of residence, and in each period of observation. Age was included as a continuous variable, and gender, birth cohorts, education, and regions were included as dummy variables ([34] and [35]).

We estimated all models using the weighted least squares (WLS) approach, as the cohorts differ in size, and when the number of observations per cell varies substantially, the disturbance term may be heteroskedastic, leading to biased standard errors. By using the WLS estimation and weighting each cell with the square root of the number of observations in each cell, we corrected for heteroskedasticity (e.g., [[49], [50], [51], [52]]).

To test the robustness of our results, we also re-ran the model by assuming that period effects on the two dependent variables derived from a business cycle effect that operates through economic conditions [53]. The results of our sensitivity analysis are shown in SubSection 3.2.

## 3 Results

Table 2 shows the results from the red and processed meat consumption-specific regression models. While the distributional characteristics of the demographic variables (age, birth cohort, region of residence), the educational level, and the average score for adherence to MD in terms of red and processed meat consumption across demographics and education have been included in the online Appendix (Tables 2A and 3A; Figs. 3A and 4A).

**Table 2:** Adherence to MD for red meat and processed meat by generation.

		*Red meat*		*Processed meat*	
		Coef.	Std.err.	Coef.	Std.err.
Age		0.0799***	0.0006	0.0117***	0.0005
*Generation (ref. Baby Boom 1)*					
Silent Generation		−0.0208**	0.0122	0.1253***	0.0115
Baby Boom 2		0.0646***	0.0111	0.0067	0.0104
Generation X		0.0964***	0.0159	0.0432**	0.0150
Male		−0.1272***	0.0058	−0.3133***	0.0054
Educational level		0.0937***	0.0063	0.1655***	0.0060
Constant		1.889***	0.0351	1.081***	0.0336
Regional Fixed Effects		YES		YES	
Observations		4398		4398	
Adj R-squared		0.5930		0.7162	

Note:  *** indicates *p* < .001, ** indicates *p* < .01 and * indicates *p* < .05.

Gender and age are confirmed to be significant demographic characteristics related to higher adherence to MD and lower consumption of red and processed meat. Women tend to follow the MD's recommended quantities for red meat and processed meat more than men do. Our results are in accordance with the previous literature, as women have more healthful food habits, while men succumb to the flavor aspects of foods and stand out for their high consumption of meat. Such differences in preferences and values influence the consumption of red and processed meat and the adherence (or lack thereof) to the Mediterranean pyramid recommendations [54]. Increasing age also leads to a higher degree of adherence to MD ceteris paribus, as older people usually eat less and focus more on their health and nutrition than younger people do because of a higher awareness and perception of risks related to their health status [55].  A higher level of education, too, appears to have a positive influence on adherence to the MD, as more educated consumers tend to follow nutritional recommendations for a healthful diet more than less educated consumers do and to know more about the relationship between a diet rich in saturated fat and cholesterol and chronic disease [56].

According to our results, the Silent Generation and the Baby Boomer 1 Generation engaged in a major shift from the traditional MD, particularly in terms of red meat consumption. Italy become more affluent after World War II, even though problems of undernutrition persisted, especially in the less developed areas of Southern Italy. Beef become more easily accessible and represented a symbol of well-being and richness, while the older generations considered the traditional MD, which was rich in plant foods like cereals, legumes, fruits and vegetables, a symbol of deprivation, rather than a healthful life choice [57].

The Baby Boomer 2 Generation adheres more closely to the MD recommendations in terms of red meat consumption, but not in terms of processed meat consumption. This generation entered early adulthood at a time of prosperity and modernization that influenced their food habits. Beef started to loss its appeal as a symbol of wealth and status, and its consumption begin to decline. The period was also characterized by social change that also affected the oldest part of the Baby Boomer 1 Generation): women entering the workforce at increasing rates (according to the ISTAT labour Force Survey (2004)“Rilevazione sulle forze di lavoro” the labor force participation rate of Italian females aged 15–64 increased of about 15% over the years 1980–1990 and of about 32% over the years1980–2000) and changes in household structures, with more single-person households that led individuals to consume more convenience food, such as ready-to-eat meals and processed meat products.

Finally, Generation X shows a lower frequency in the consumption of red and processed meat. Generation X entered early adulthood in an era characterized by an overall decline in the consumption of red and processed meat in favor of more sustainable and healthful foods. In 2001, the first domestic BSE case in Italy was also detected, leaving creating a shadow on the safety of beef. These concerns lead to a significant drop in beef consumption in that period an increased lack of confidence in the wisdom of consuming beef products.

### 3.1 Dietary transition and geographic differences

Italy is characterized by complex regional dietary patterns with significant geographic differences in culinary traditions, as well as in culture, economic development and in climate (with the South with a very warm weather and the North with a cold one for a large portion of the year) that have affected attitudes toward certain foods [58].

To determine the presence of a North-South gradient in the adherence to the MD diet pyramid's recommendations and in the evolution in red and processed meat consumption across generations, we re-ran the model on two subsamples using geographic dummies for the North and South of Italy. The results are shown in Tables 3 and 4.

**Table 3:** Adherence to MD for red meat by generation: north-south gradient.

		*Noth Regions*		*South Regions*	
		Coef.	Std. Err.	Coef.	Std. Err.
Age		0.0083***	0.0006	0.0068***	0.0006
*Generation (ref. Baby Boom 1)*					
Silent Generation		−0.0298*	0.0122	0.008	0.0121
Baby Boom 2		0.0921***	0.0111	0.0217*	0.0106
Generation X		0.0948***	0.0159	0.0795***	0.0154
Male		−0.1801***	0.0058	−0.0875***	0.0057
Educational level		0.1404***	0.0063	0.0318***	0.0063
Constant		1.8736***	0.0351	1.9527***	0.0339
Regional Fixed Effects		YES		YES	
Observations		1666		1803	
Adj R-squared		0.7149		0.4201	

Note:  *** indicates *p* < .001, ** indicates *p* < .01 and * indicates *p* < .05.

**Table 4:** Adherence to MD for processed meat by generation: North-South Gradient.

		*North Regions*		*South Regions*	
		Coef.	Std. err.	Coef.	Std. err.
Age		0.0108***	0.0008	0.0114***	0.0008
*Generation (ref. Baby Boom 1)*					
Silent Generation		0.0420*	0.0166	0.2532***	0.0166
Baby Boom 2		0.0491**	0.0151	−0.0613***	0.0146
Generation X		0.0726**	0.0216	−0.0367	0.0212
Male		−0.3233***	0.0079	−0.3116***	0.0078
Educational level		0.1992***	0.0086	0.1166***	0.0086
Constant		1.117***	0.0477	1.518***	0.0464
Regional Fixed Effects		YES		YES	
Observations		1666		1803	
Adj R-squared		0.7307		0.7960	

Note:  *** indicates *p* < .001, ** indicates *p* < .01 and * indicates *p* < .05.

Our findings support the presence of a North-South gradient in the consumption of red meat, as expected, with residents of the North consuming red meat more frequently than those of the South. Our results also revealed standardization in the evolution of red meat consumption across generations in the country's two geographical areas. As for the full sample, the oldest generations (the Silent and Baby Boomer 1 Generations) in the two macro-areas present similar dietary lifestyles that are characterized by greater frequency of red meat consumption and lower scores on the MD pyramid recommendations. The younger generations, again, seem to be more oriented to lower consumption of red meat than the older generations are.

The analysis of our results on the evolution of processed meat consumption across the two macro-areas and across generations, however, indicates a more complex pattern with differences that reflect the dissimilar “food culture” and economic conditions that have characterized the Italian regions in the last several decades. The northern regions again consume processed meat more often than the southern ones, and it is the Baby Boomer 1 Generation that has the lowest degree of compliance with the MD diet. In the diffusion process of processed meat, the southern regions appear to lag one generation behind the northern regions, as those in the South who present the lowest adherence to the MD, ceteris paribus, are the Baby Boomer 2 Generation and Generation X.

Arguably, the economic and social dualism between the more economically developed northern regions and the less developed southern regions may have interacted with the diffusion of convenience food more than with the diffusion of red meat [21,59]. Both Baby Boomer generations enjoyed the postwar economic development, which might have influenced their food choices. Women began to be a steady presence in Italy's work force, and the time they had to spend in preparing food declined, creating a progressive entry of processed foods on Italian market that conquered a broader segment of consumers in the northern regions. While in the North the two Baby Boomer generations started to became “convenience seekers,” with increasing preferences for foods that require less time to be prepared, the less developed South remained tied to traditional cuisine and meal preparation, which was still considered an important activity.

The “traditional” pattern that was a peculiar characteristic of the oldest generations of the South was abandoned later by the younger generations in favor of a rise preferences for processed products like processed meat. While Generation X in Northern Italy was more oriented toward sustainable and healthful foods, the same generation in the South became the new “convenience seeker,” with food habits farther from those recommended by the MD pyramid [60].

According to our results, the Silent and Baby Boomer 1 Generations are characterized by a major shift from the traditional MD in terms of red meat consumption, which these generations saw as a symbol of prosperity, while the Baby Boomer 2 Generation, especially in northern Italy, had a tendency to consume more convenience food, such as processed meat, in response to their need for meals that could be prepared quickly.

The health, social and environmental concerns that are associated with red and processed meat consumption are part of an ongoing global debate and have led younger generations to adopt more healthful and environmentally sustainable eating patterns. However, despite the increasing shift away from the consumption of red meat products among the youngest generations, our findings echo previous findings regarding older age's association with poor health, as the evolution in the oldest generations diet toward more animal products has increased their risk of developing chronic health conditions in later life, placing an increasing burden on the National Health Service [61].

According to our findings, a country's eating regime is deeply rooted in its history and dietary patterns are shaped by consumers’ culture, context, and socioeconomic status. Therefore, altering individuals' consumption decisions is often challenging. Informational campaigns should consider that, among the oldest generations, health-related reasons for consuming less red and processed meat may be perceived as more convincing than environmental reasons are likely to be [62].

### 3.2 Sensitivity analysis

In order to test the robustness of our results, we also re-run the model by assuming that period effects on the red and processed meat consumption could derive from a business cycle effect operating through economic conditions. Indeed, Clark et al. (2010) show that changes in aggregate economic conditions affect subjective well-being, which may also influence the propensity towards red and processed meat consumption [53].

We include in the regression model the GDP growth rate (see Table 5). Even though the inclusion of period effects, which derive from business cycle fluctuations, lowered the precision of the estimates of the age and cohort effects, the patterns of the age and cohort profiles remain similar.

**Table 5:** Adherence to MD for red meat and processed meat by generation and GDP growth rate.

		*Red meat*		*Processed meat*	
		Coef.	Std. Err.	Coef.	Std. Err.
Age		0.0061***	0.0005	.0112***	0.0007
*Generation (ref. Baby Boom 1)*					
Silent Generation		0 0.0081	0.0099	0.1335***	0.0144
Baby Boom 2		0.0522***	0.0087	0.0098	0.0126
Generation X		0.0647***	0.0131	0.0384*	0.0190
Male		−0.1286***	0.0044	−0.3126	0.0064
Educational level		0.0972***	0 0.0048	0.1638***	0.007
GDP growth rate		−0.0063	0.0039	−0.0001	0.0056
Constant		1.9732	0.0297	1.1011***	0.0431
Regional Fixed Effects		YES		YES	
Observations		3242		3242	
Adj R-squared		0.5863		0.7142	

Note: *** indicates *p* < .001, ** indicates *p* < .01 and * indicates *p* < .05.^1^.

## 4 Conclusions and policy implications

The choice of foods as part of a diet implies a complex mechanism of interactions between social and cultural processes, values and traditions [63]. Our results revealed that increases in disposable income, changes in women's role in society, and urbanization and globalization have had significant effects on the Mediterranean lifestyle. Some of the more important of these social changes came to Italy in the second half of the last century, when the Silent and Baby Boomer Generations entered early adulthood, profoundly affecting their adherence to the MD and leading to progressive abandonment of traditional food habits in favor of consumption of animal protein, particularly of red and processed meat.

Arguably, more stringent regulation is needed to reduce red and processed meat consumption. These meats could be treated like other carcinogens and foods that raise public health concerns. For instance, informational campaigns could be combined with pricing instruments like “sin” taxes—designed according to the Pigouvian principle—that incorporate the health and environmental costs of meat consumption into the price paid by the consumers, forcing them to take the outcomes of their consumption habits into account when they choose what foods to consume [[64], [65], [66], [67], [68]]. School-based food and nutrition education interventions, with actions such as change in school meals, educational posters by the school, organization of events, can increase the health and environmental consciousness of adolescents and would be advisable too [69]. The combination of such policies should be effective in encouraging consumers to swap red and processed meat consumption for a plant-based diet like the MD, whose principles could be an important step toward a more sustainable future [70].

Our study presents a limitation: since it relies on a pseudo-panel approach we cannot take explicit account of individual-specific heterogeneity in red and processed meat consumption since it involves aggregation into prototypes of individuals (i.e., cohorts) identified by characteristics that remain stable over time. Despite this limitation, our approach is particularly suitable to investigate how food consumption habits of different demographic groups change over time or due to aggregate changes in culture, economic conditions, and environmental and health concerns.

## Authors’ contributions

The interpretation and reporting of the results are the sole responsibility of the authors.

All authors contributed to the conception of the study, the design of the study, and writing of the manuscript. All the authors commented on/edited all drafts of the manuscript.

All the authors can be identified as the guarantors for the overall content and interpretation of the results.

## Funding

None.

## Declaration of Competing Interest

The authors report no declarations of interest.

The views expressed are those of the authors and do not necessarily reflect opinions of the institutions of affiliation.

## Appendix Supplementary materials

Image, application 1
