# Supplementary material for: Improving health and sustainability: Patterns of red and processed meat consumption across generations
Source: Health Policy. 2022 Dec;126(12):1324–30. doi: 10.1016/j.healthpol.2022.10.006 (PMC9709574; doi:10.1016/j.healthpol.2022.10.006)

**Online Appendix**

| Table 1A: Medium age at which women left their parental household | | | |
| --- | --- | --- | --- |
| *Generations* | *Medium Age* | *Period* |  |
| Silent | 23 | Between the ’50 and the end of ’60 |  |
| Baby Boomer 1 | 23 | Between the ’70 and the’80 |  |
| Baby Boomer 2 | 24 | Between the ’80 and the’90 |  |
| Generation X | 25 | Between the ’90 and the beginning of 2000 |  |

Source: ISTAT, 2014.

Figure 1A: Per capita Consumption Trends Bovine Meat since 1961 – Italy


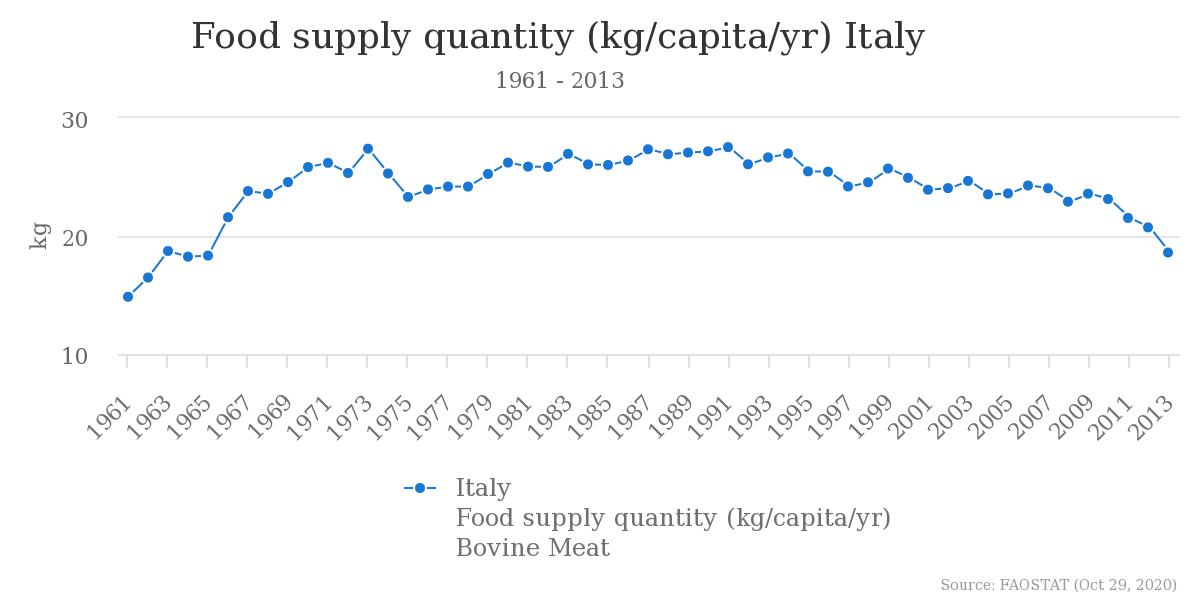


Source: FAO – meat supply data (potentially available for human consumption).

Figure 2A: Per capita Consumption Trends Bovine Meat since 1961 – Italy, Southern Europe, Europe Union


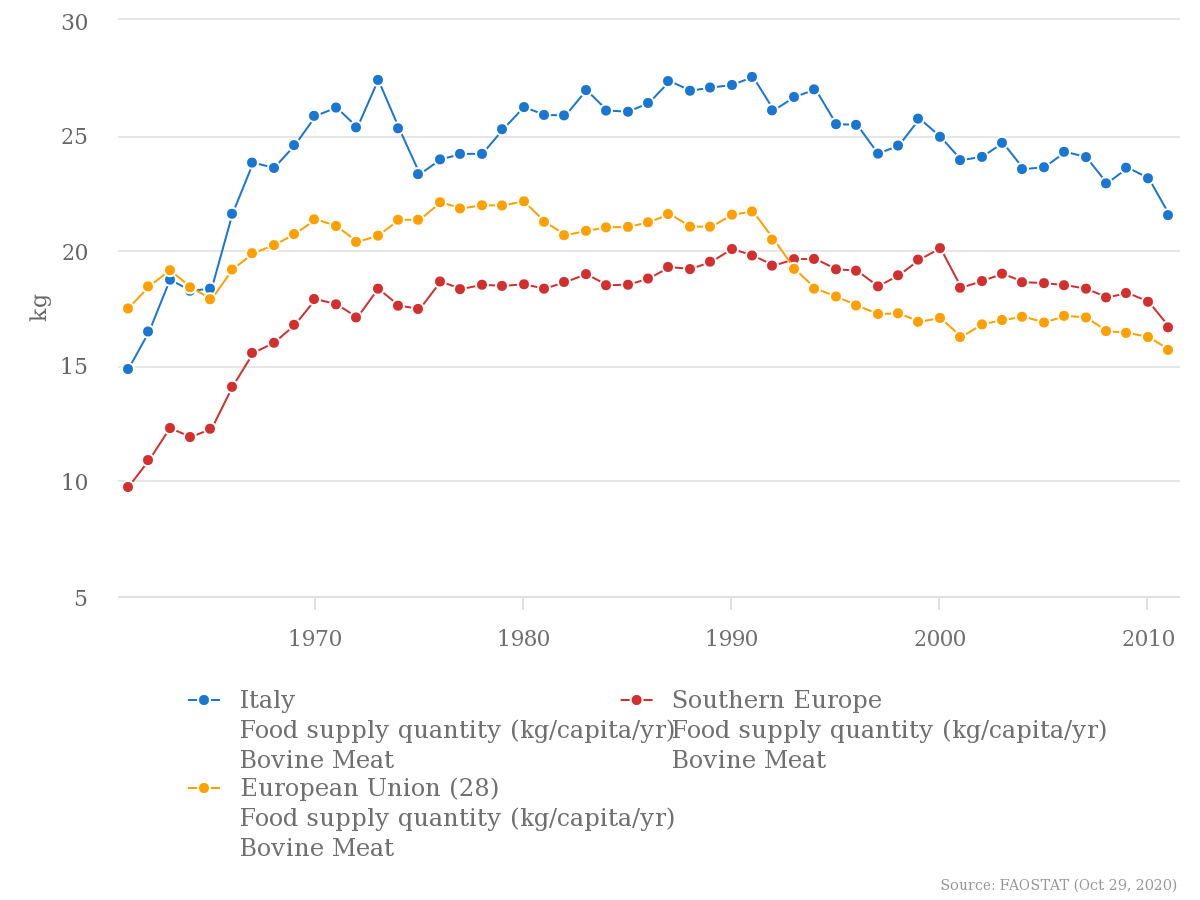


Source: FAO – meat supply data (potentially available for human consumption).

Table 2A: Size Distribution of cells (1997-2012)

| *Sample* | *Mean* | *Min (*)* | *Max* |
| --- | --- | --- | --- |
| Italy | 128 | 31 | 768 |
| Northern Regions | 143 | 31 | 768 |
| Southern Regions | 121 | 31 | 728 |

(*) Cells with 30 or fewer individuals were excluded from the analysis to ensure robust estimates of the subgroups’ mean statistics (see Verbeek, 2008).

Table 3A: Red meat: mean values of the score by generations, age, education, regions and gender (18 years and over): 1997-2012

|  | Mean value of the score by Generation | | | | Population distribution (%) | | | |
| --- | --- | --- | --- | --- | --- | --- | --- | --- |
|  | Silent  Generation | Baby Boomer 1 | Baby Boomer 2 | Generation X | Silent  Generation | Baby Boomer 1 | Baby Boomer 2 | Generation X |
| *Age* |  |  |  |  |  |  |  |  |
| 18-34 |  |  | 2.2772 | 2.2210 |  |  | 5.20 | 67.22 |
| 35-49 |  | 2.2532 | 2.2889 | 2.2862 |  | 27.23 | 79.19 | 32.77 |
| 50-64 | 2.3108 | 2.3160 | 2.3674 |  | 35.68 | 71.18 | 15.59 |  |
| 65+ | 2.4090 | 2.4679 |  |  | 64.32 | 1.58 |  |  |
| *Educational level* |  |  |  |  |  |  |  |  |
| Low | 2.3695 | 2.2681 | 2.2454 | 2.1960 | 83.33 | 61.23 | 48.51 | 37.05 |
| High | 2.4141 | 2.3593 | 2.3543 | 2.2747 | 16.67 | 38.77 | 51.49 | 62.95 |
| *Regions* |  |  |  |  |  |  |  |  |
| North | 2.3940 | 2.3337 | 2.3560 | 2.2794 | 44.33 | 42.37 | 42.07 | 41.27 |
| Centre | 2.2741 | 2.1993 | 2.1990 | 2.1372 | 19.60 | 18.60 | 18.18 | 17.63 |
| South | 2.4201 | 2.3250 | 2.2882 | 2.2597 | 36.05 | 39.02 | 39.74 | 41.09 |
| *Sex* |  |  |  |  |  |  |  |  |
| Female | 2.4294 | 2.3402 | 2.3550 | 2.3440 | 53.22 | 50.88 | 50.92 | 50.54 |
| Male | 2.3162 | 2.2666 | 2.2485 | 2.1477 | 46.77 | 49.11 | 49.07 | 49.45 |

Table 4A: Processed meat: mean values of the score by generations, age, education, regions and gender (18 years and over): 1997-2012.

|  | Mean value of the score by Generation | | | | Population distribution (%) | | | |
| --- | --- | --- | --- | --- | --- | --- | --- | --- |
|  | Silent  Generation | Baby Boomer 1 | Baby Boomer 2 | Generation X | Silent  Generation | Baby Boomer 1 | Baby Boomer 2 | Generation X |
| *Age* |  |  |  |  |  |  |  |  |
| 18-34 |  |  | 1.7823 | 1.6494 |  |  | 5.20 | 67.22 |
| 35-49 |  | 1.7672 | 1.7529 | 1.7504 |  | 27.23 | 79.19 | 32.77 |
| 50-64 | 1.9678 | 1.8977 | 1.8733 |  | 35.68 | 71.18 | 15.59 |  |
| 65+ | 2.1774 | 2.0154 |  |  | 64.32 | 1.58 |  |  |
| *Educational level* |  |  |  |  |  |  |  |  |
| Low | 2.0976 | 1.8037 | 1.6832 | 1.5946 | 83.33 | 61.23 | 48.51 | 37.05 |
| High | 2.1683 | 1.9694 | 1.8599 | 1.7421 | 16.67 | 38.77 | 51.49 | 62.95 |
| *Regions* |  |  |  |  |  |  |  |  |
| North | 1.9360 | 1.7914 | 1.7487 | 1.6619 | 44.33 | 42.37 | 42.07 | 41.27 |
| Centre | 2.1793 | 1.9319 | 1.8543 | 1.8053 | 19.60 | 18.60 | 18.18 | 17.63 |
| South | 2.3380 | 1.9389 | 1.7653 | 1.6556 | 36.05 | 39.02 | 39.74 | 41.09 |
| *Sex* |  |  |  |  |  |  |  |  |
| Female | 2.2258 | 1.9978 | 1.9162 | 1.8859 | 53.22 | 50.88 | 50.92 | 50.54 |
| Male | 1.9738 | 1.7357 | 1.6328 | 1.4902 | 46.77 | 49.11 | 49.07 | 49.45 |

Figure 3A : Red meat consumption by generation: mean scores of MD adherence. (The maps were constructed by taking for each generation the average scores over the period 1997–2012)


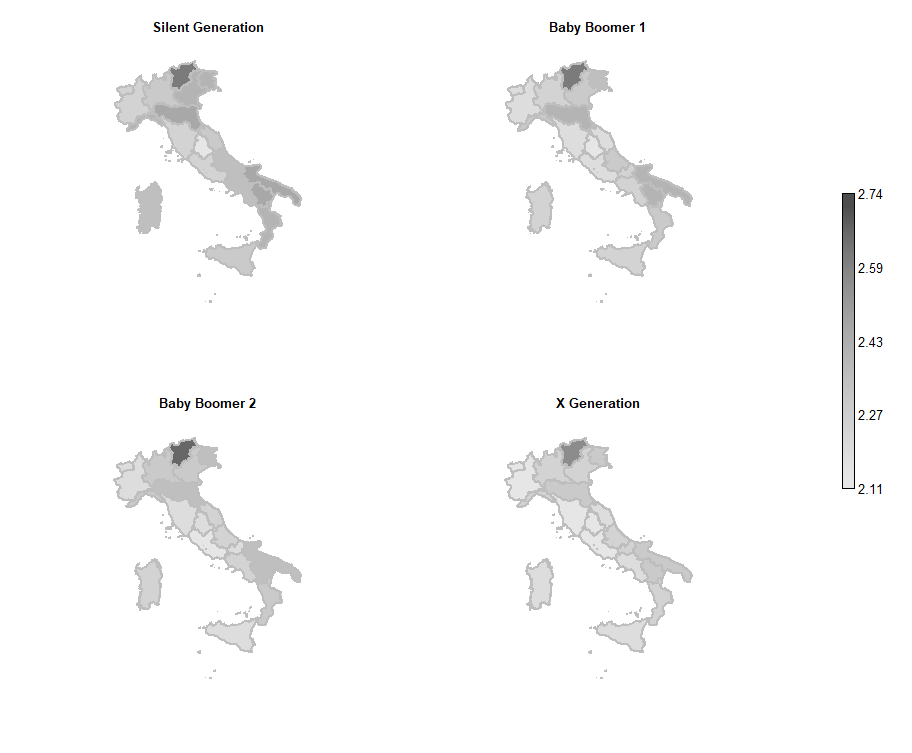


Figure 4A: Processed meat consumption by generation: mean scores of MD adherence. (The maps were constructed by taking for each generation the average scores over the period 1997–2012.)


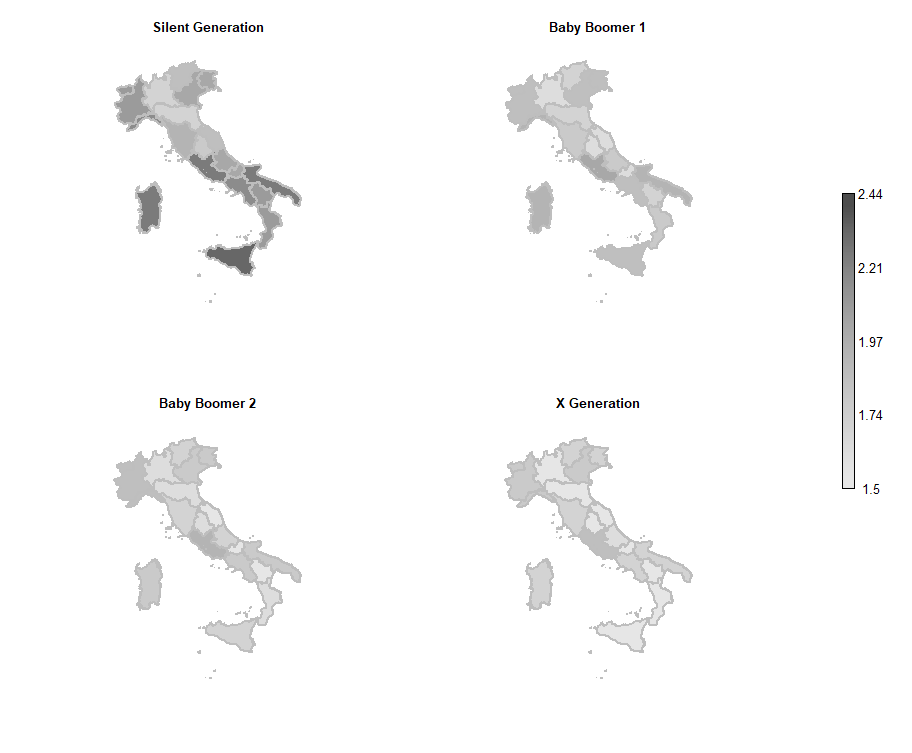

Supplement: Supplementary file 1 [file mmc1.docx]
